# Efficacy of interval exercise training to improve vascular health in sedentary postmenopausal females

**DOI:** 10.14814/phy2.15441

**Published:** 2022-08-19

**Authors:** Gemma K. Lyall, Gurpreet K. Birk, Emma Harris, Carrie Ferguson, Kirsten Riches‐Suman, Mark T. Kearney, Karen E. Porter, Karen M. Birch

**Affiliations:** ^1^ School of Biomedical Sciences, Faculty of Biological Sciences and Multidisciplinary Cardiovascular Research Centre, University of Leeds Leeds UK; ^2^ IVS Ltd, Vascular Ultrasound, Royal Oldham Hospital Oldham UK; ^3^ Vascular Ultrasound, Radiology, Leeds General Infirmary Leeds UK; ^4^ School of Human and Health Sciences, Centre for Applied Research in Health University of Huddersfield Huddersfield UK; ^5^ Institute of Respiratory Medicine and Exercise Physiology, Rehabilitation Clinical Trials Center, Division of Respiratory and Critical Care Physiology and Medicine The Lundquist Institute for Biomedical Innovation at Harbor‐UCLA Medical Center Torrance California USA; ^6^ School of Chemistry and Biosciences University of Bradford Bradford UK; ^7^ Leeds Institute of Cardiovascular and Metabolic Medicine & Multidisciplinary Cardiovascular Research Centre, University of Leeds Leeds UK

**Keywords:** circulating angiogenic cells, endothelial function, interval exercise, postmenopausal females, V̇O_2peak_

## Abstract

**Background:**

Menopause represents a turning point where vascular damage begins to outweigh reparative processes, leading to increased cardiovascular disease (CVD) risk. Exercise training reduces CVD risk in postmenopausal females via improvements in traditional risk factors and direct changes to the vasculature. We assessed the effect of moderate (MODERATE‐IT) versus heavy (HEAVY‐IT) intensity interval exercise training upon markers of cardiovascular health and vascular repair in postmenopausal females.

**Methods:**

Twenty‐seven healthy postmenopausal females (56 ± 4 yr) were assigned to 12 weeks of either MODERATE‐IT or HEAVY‐IT, twice per week. MODERATE‐IT consisted of 10s work, and 10s active recovery repeated for 30 min. HEAVY‐IT comprised 30s work, and 30s active recovery repeated for 21 ± 2 min. Endothelial function (flow‐mediated dilation), arterial stiffness (pulse wave velocity), and V̇O_2peak_ were assessed pre‐training and post‐training. Blood samples were obtained pre‐training and post‐training for enumeration of circulating angiogenic cells (CACs), culture of CACs, and lipoprotein profile.

**Results:**

V̇O_2peak_ increased 2.4 ± 2.8 ml/kg/min following HEAVY‐IT only (*p* < 0.05). Brachial blood pressure and endothelial function were unchanged with exercise training (*p* > 0.05). Peripheral pulse wave velocity reduced 8% with exercise training, irrespective of intensity (*p* < 0.05). Exercise training had no effect on lipoprotein profile or endothelin‐1 (*p* > 0.05). CAC adhesion to vascular smooth muscle cells (VSMC) increased 30 min post plating following MODERATE‐IT only (*p* < 0.05).

**Conclusions:**

HEAVY‐IT was more effective at increasing V̇O_2peak_ in postmenopausal females. The ability of CACs to adhere to VSMC improved following MODERATE‐IT but not HEAVY‐IT. Interval training had the same effect on endothelial function (no change) and arterial stiffness (reduced), regardless of exercise intensity.

## 1 INTRODUCTION

Cardiovascular disease (CVD) remains a leading cause of morbidity and mortality, with age a significant CVD risk factor (BHF, 2020). Prior to the menopause, females show a 7–10 years lag in CVD development compared to males (Burns & Korach, 2012). However, following the menopause, the rate of decline in cardiovascular health is faster in females versus males, resulting in narrowing of the sex difference in rates of CVD (Maas & Appelman, 2010). Traditional CVD risk factors (i.e., V̇O_2peak_, blood pressure [BP], obesity, lipoproteins, etc.) account for 40%–60% of CVD risk, with the remaining CVD risk hypothesized to be related to changes in the vascular environment (Green et al., 2008; Joyner & Green, 2009; Mora et al., 2007; Thijssen et al., 2010). Therefore, modifying both the vasculature and traditional risk factors, such as V̇O_2peak_, is integral for reducing overall CVD‐related morbidity and mortality in postmenopausal females.

Estrogen has an important role in female cardiovascular health (see Novella et al. (2019)). Reductions in estrogen following menopause contributes to decreased nitric oxide (NO) production and bioavailability, through reduced endothelial nitric oxide synthase (eNOS) synthesis and increased production of superoxide anions (Lekontseva et al., 2010). This decrease in NO bioavailability reverses inhibition of endothelin‐1 (ET‐1) production leading to increased basal vasoconstriction (Lekontseva et al., 2010). Additionally, lower circulating estrogen favors collagen over elastin production in the vasculature (Natoli et al., 2005). Greater collagen production combined with higher pulse pressure as a result of aging contributes to greater arterial stiffness (Zieman et al., 2005). Thus, the combination of menopause and aging is associated with endothelial dysfunction and arterial stiffening, at a time when estrogen loss impairs the capacity for repair within the endothelium, through reductions in the number and function of circulating angiogenic cells (CACs) (Fadini et al., 2008). In healthy vasculature, CACs migrate to areas of endothelial damage to aide repair via secretion of proangiogenic cytokines and growth factors. Impaired capacity for repair, as indicated by fewer colony‐forming units (CFUs), has been associated with poorer brachial artery endothelial function, which in turn has been associated with increased risk of coronary artery disease (Hill et al., 2003; Kunz et al., 2006).

Previous studies investigating different types of exercise interventions, that is, interval versus continuous, at differing intensities, for example, low, moderate, high, sprint, and vigorous, in postmenopausal females have shown mixed results in relation to changes in cardiovascular health. This lack of clarity in defining exercise intensity may, in part, be responsible for the disparate findings in regard to physiological variables assessed in the studies. When high‐intensity aerobic exercise (Hoier et al., 2021) or sprint interval exercise (Ho et al., 2020) was used in an exercise intervention, cardiorespiratory fitness improved (Ho et al., 2020; Hoier et al., 2021; Swift et al., 2014). In contrast, exercise at low, moderate, and vigorous intensity improved arterial stiffness in postmenopausal females, suggesting exercise intensity may not be a critical factor (Ho et al., 2020; Sugawara et al., 2004; Sugawara et al., 2006). However, improvements in endothelial function have appeared less responsive to exercise interventions at any exercise intensity in postmenopausal females (Ho et al., 2020; Hoier et al., 2021), unless pre‐training endothelial function was impaired (Swift et al., 2014). It remains unclear what type of exercise and indeed the intensity of exercise is most beneficial for improvements in cardiovascular health in postmenopausal females, particularly for endothelial function and markers of vascular repair.

We have reported that following a single session of exercise in postmenopausal females, the number of CACs remained constant, while CFU number increased to the same extent following both moderate and heavy‐intensity interval exercise, but not moderate continuous exercise (Harris et al., 2017). This is intriguing as exercise intensity is known to be important for other aspects of cardiovascular health, that is, V̇O_2peak_, with the intensity of exercise shown to be inversely correlated with risk of cardiovascular events in postmenopausal females (Manson et al., 2002). While exercise intensity appeared to be unimportant for increasing markers of vascular repair following a single session of interval exercise (Harris et al., 2017), other cardiovascular markers may be increased to a greater extent with heavy‐intensity interval exercise following training, which is yet to be investigated in postmenopausal females. Furthermore, while heavy‐intensity interval exercise may not have a greater effect on markers of vascular repair compared to moderate‐intensity interval exercise (Harris et al., 2017), a greater increase in V̇O_2peak_ following heavy‐intensity interval training would indicate that heavy‐intensity interval training is more effective for overall reduction in CVD risk. This is particularly relevant in sedentary populations where lack of physical activity is associated with increased CVD risk (Bellettiere et al., 2019). Therefore, in the present study, we used the most effective acute exercise protocols from the previous Harris et al. (Harris et al., 2017) study, that is, the moderate and heavy‐intensity interval exercise sessions, and evaluated their effectiveness on cardiovascular health markers in postmenopausal females with elevated CVD risk due to sedentary behavior and menopausal reductions in estrogen over the course of 12 weeks of exercise training.

Therefore, the aim of the present study was to compare the effect of two different interval exercise training protocols of differing exercise intensities (moderate versus heavy) upon traditional CVD risk factors (i.e., BP, lipoproteins, V̇O_2peak_) and markers of vascular health and repair (endothelial function, arterial stiffness, CAC number, and function) in sedentary postmenopausal females. We hypothesized that heavy‐intensity interval training (HEAVY‐IT) would be more effective at improving traditional CVD risk factors and markers of vascular health and repair, compared to moderate‐intensity interval training (MODERATE‐IT) in postmenopausal females.

## 2 METHODS

### 2.1 Participants

Recruitment yielded 65 potential participants for screening, of which 31 were successfully screened and invited for testing. Reasons for screening failures included 12 on medications, 1 scheduled for surgery, 2 physically active, 1 smoker, and 18 unavailable for the duration of testing. Two participants dropped out prior to baseline visits, with a further participant dropping out during training and one participant suffering an unrelated broken leg prior to post testing. This resulted in 27 healthy, sedentary females (56 ± 4 years) successfully completing the study. Participants were 6 ± 3 years post menopause, defined as absence of menstrual cycle, free of self‐reported current or previous risk factors associated with cardiovascular/ respiratory diseases and metabolic disorders. Participants were non‐smokers and were not taking medication, including hormone replacement therapy (HRT). Informed consent was gained from all participants, and the study procedures were approved by the University of Leeds Ethics Committee and adhered to the Declaration of Helsinki.

### 2.2 Experimental protocol

Participants visited the laboratory twice prior to and twice following a 12‐week exercise training intervention. The first visit consisted of brachial blood pressure (BP), brachial artery endothelial function via flow‐mediated dilation (FMD), arterial stiffness via pulse wave velocity (PWV), and venous blood sampling from the antecubital fossa for enumeration of circulating angiogenic cells (CACs), culture of CACs, and analysis of the lipoprotein profile and endothelin‐1 (marker of vasoconstriction). All assessments were conducted in a quiet, temperature‐controlled environment. Participants were asked to fast (including vitamin intake) for >8 h, abstain from alcohol and caffeine for 16 h, and not to perform any exercise for 24 h prior to assessments. During the second visit (2–4 days later), on a separate day, height, weight, and waist–hip ratio (WHR) were measured, and a ramp‐incremental step exercise test (RISE) to intolerance was performed to measure peak oxygen uptake (V̇O_2peak_). Prior to this visit, participants were asked to abstain from alcohol and caffeine for 16 h, and not to perform any exercise for 24 h prior to assessments. Participants were then matched for age, BMI, and V̇O_2peak_, and randomly assigned to either MODERATE‐IT (*n* = 13) or HEAVY‐IT (*n* = 14). Following 12 weeks of exercise training, all pre‐training assessments were repeated over two separate sessions, between 48 and 72 h after the last exercise training session.

### 2.3 Ramp‐incremental step exercise (RISE) test

Participants performed a RISE to directly measure V̇O_2peak_, before, at 6 weeks and after 12 weeks of exercise training on a cycle ergometer (Excalibur Sport V.2.0; Lode BV), as described in detail previously (Harris et al., 2014; Rossiter et al., 2006). Breath by breath pulmonary gas exchange (Medgraphics, Medical Graphics Corporation) was measured during the RISE test and used to estimate the gas exchange threshold (GET), and measure V̇O_2peak_. The V̇O_2peak_ of both ramp and step phases was calculated using an 8‐point rolling average over 12 breaths taken at the end of both phases; comparison of V̇O_2peak_ during the ramp and step phases was used to confirm V̇O_2max_ (Bowen et al., 2012). GET was determined from the ramp portion of the RISE test and estimated using the V‐slope method (Wasserman et al., 1999). This was then used to determine the work rates required for exercise training in either the moderate‐intensity domain (below the GET) or the heavy‐intensity domain (above the GET). Following reassessment of GET and V̇O_2peak_ at 6 weeks of exercise training, work rates were adjusted as necessary.

### 2.4 Exercise training protocols

Exercise training consisted of two laboratory‐based cycle ergometry sessions per week, for 12 weeks, with a single unsupervised exercise training session conducted at home every week. This was to reflect the American College of Sports Medicine physical activity guidelines of 20 min of high‐intensity physical activity three times per week (HEAVY‐IT group) or 30 min of moderate exercise five times per week. We prescribed the MODERATE‐IT group only three 30 min sessions per week to match the total work of the HEAVY‐IT group. The MODERATE‐IT group completed repeated intervals of 10 s work and 10 s active recovery (20 W) for 30 min at a work rate that evoked a V̇O_2_ equivalent to 90 ± 10% GET, that is, moderate‐intensity exercise domain (Manson et al., 2002). The HEAVY‐IT group completed repeated intervals of 30 s work and 30 s active recovery (20 W), with work bouts conducted at a work rate that evoked a V̇O_2_ equivalent to 120 ± 10% GET, that is, heavy‐intensity exercise domain (Whipp, 1996). The duration of HEAVY‐IT sessions, mean 21 ± 2 min, was altered for each participant randomized to the HEAVY IT group to match both groups for total work based on energy expenditure (kJ). Previous studies have suggested that energy expenditure rather than exercise intensity may be more influential for improvements in markers of cardiovascular health in postmenopausal females (Sugawara et al., 2004), therefore by matching energy expenditure between the two exercise groups, the effect of exercise intensity upon the cardiovascular markers assessed in the present study could be delineated. Exercise intensity was confirmed using breath‐by‐breath gas analysis during the first exercise training session and reassessed at 6 weeks of training following a midpoint RISE test, as described above.

Participants were instructed to conduct home‐based sessions for the same duration and in an equivalent manner to that of the supervised sessions using cycling, walking, swimming, or jogging activity. During the supervised exercise sessions, participants were asked to state their rate of perceived exertion (RPE) and attempt to approximate the same RPE during their home‐based sessions.

### 2.5 Assessment of brachial artery endothelial function using FMD

Assessment of brachial artery endothelial function was performed in accordance with previous guidelines for FMD (Thijssen et al., 2011), with a coefficient of variation for FMD in our lab of 15% (Rakobowchuk, Harris, Taylor, Baliga, et al., 2012). Resting brachial artery diameter was assessed in the distal third of the upper right arm for 20s following a 20‐min rest period using a 7 MHz linear array ultrasound probe (Aspen, Acuson, Siemens Medical). A blood pressure cuff was then immediately inflated on the right forearm, distal to the ultrasound probe, to >200 mmHg for 5 min. Brachial artery diameter and blood flow velocity were recorded at 20 frames per second for 30s prior to cuff deflation and continued for 150 s following cuff deflation using Vascular Imager software (Medical Imaging Applications). These images were then analyzed using semi‐automatic edge detection software (Brachial Tools V.5, Medical Imaging Applications). Peak reactive hyperemia, peak shear rate, and area under the shear rate curve from cuff release to 60 s (AUC_60_) and 90 s (AUC_90_) post cuff release were calculated, as previously described (Rakobowchuk, Harris, Taylor, Baliga, et al., 2012). The insonation angle for blood flow velocity measurement was 60 ± 2° for all measurements.

Following analysis of FMD results, participants were split into two separate categories of normal FMD and impaired FMD as described in Rossi et al. (Rossi et al., 2008). Relative FMD values prior to training and irrespective of exercise training group were used to split participants into normal FMD (>4.5%) or impaired FMD (<4.5%). The effect of exercise training upon FMD was then determined (Delta FMD), within the normal and impaired FMD groups. Subsequently, normal and impaired FMD were also split into MODERATE‐IT and HEAVY‐IT to determine whether one type of exercise training was more beneficial for changing the baseline FMD in those in whom this is impaired.

### 2.6 Assessment of arterial stiffness

Arterial stiffness was assessed via applanation tonometry (SphygmoCor system, SCOR‐Vx, AtCor Medical Pty Ltd) between the carotid‐radial, carotid‐brachial arteries and between the brachial‐foot, previously described in detail (Rakobowchuk, Harris, Taylor, Cubbon, & Birch, 2012). Briefly, a handheld pressure transducer was held perpendicular to the arterial site of interest (carotid, brachial, radial, foot) to acquire 20 to 40 arterial blood pressure waveforms at each arterial site. The distance between the sternal notch and the arterial site of interest was measured while participants were in a supine position with simultaneous ECG recording and outputted with low‐pass filter to LabChart 7.0 (ADInstruments). PWV was calculated as:

$$\mathrm{PWV}\;\left(m/s\right)=\text{∆distance}/\text{∆PTT}\;$$

where ∆distance is the difference in distance between the sternum and the two arterial sites, and ∆PTT is the difference in pulse transit time between the two sites. The PTT at each arterial site was calculated as the time delay between the peak of the R‐wave on the ECG and the foot of the upstroke on the pulse pressure waveform when acquired simultaneously.

### 2.7 Assessment of lipoprotein profile and endothelin‐1

Blood samples (EDTA vacutainers) were immediately centrifuged at 3000 rcf for 10 min at 4°C to yield plasma which was stored at −80°C until required for analysis. Samples were analyzed for triglyceride, total cholesterol, LDL, and HDL concentrations by local hospital pathology services (Cobas C311 system, Hoffman, La Roche). Endothelin‐1 (ET‐1) concentrations were subsequently determined from stored plasma samples via ELISA (R&D Systems, Minneapolis), as per the manufacturer's guidelines. All standards and samples were tested in duplicate with a correlation of >0.99 achieved for curve estimation in all samples.

### 2.8 Circulating angiogenic cell (CAC) number and assessment of adhesive function

Fasting venous blood samples were collected in EDTA vacutainers. Peripheral blood mononuclear cells were harvested from whole blood using Ficoll gradient centrifugation as we described previously (Cubbon et al., 2014). Purification of CAC was achieved by fluorescence‐activated cell sorting, followed by magnetic separation of CD34^+^ cells, those specifically acknowledged as CACs, from 10 ml of blood, pre‐exercise and post‐exercise training, using a commercially available kit (Miltenyi Biotec) as previously described (Rakobowchuk, Harris, Taylor, Baliga, et al., 2012). In the current study, CACs were defined as double positive (CD34^+^/CD45^dim^) or triple positive (CD34^+^/CD45^dim^/KDR+) and enumerated as a percentage of the total number of leukocytes.

To provide CAC for functional assays, cells were cultured at a density of 5 × 10^6^/well in fibronectin‐coated plates in endothelial growth medium (EGM; endothelial basal medium supplemented with 20% fetal cell serum, growth factors, and antibiotics; EBM‐2 Bullet kit; Lonza, Inx). Cells were cultured in a humidified incubator at 37°C in 5% CO_2_ in air for 7 days (changing medium on days 2, 4, and 6), after which adherent cells were released using trypsin/EDTA, centrifuged at 300 *g* for 10 min, and 5 × 10^6^ cells were resuspended in 2 ml of EndoCult growth medium (StemCell Technologies).

In parallel with these cultures, human vascular smooth muscle cells (VSMC), harvested and cultured as we described previously (Porter et al., 2002), were expanded in culture, seeded (2 × 10^4^ per well) into 96‐well plates, and grown to confluence (1–2 days). VSMC medium was removed, VSMC washed with PBS, and harvested. CAC suspensions were then added to the preformed monolayers of human vascular smooth muscle cells, in duplicate wells at a density of 2.5 × 10^4^/well in 100 μl of EGM. The resultant cell co‐cultures were returned to incubation and microscopic images at 100× magnification (five randomly selected images per well) allowed the number of adherent CACs to the VSMC monolayers to be counted after 10, 20, 30, 60 min, and 24 h. Non‐adherent CACs were aspirated, adherent cells were washed gently with PBS, and then, 4% paraformaldehyde (100 μl/well) was added to each well to fix, apart from the 24 h wells to allow for any further adhesion or cell loss to be quantified beyond the 1‐h time point. Adherent CACs were imaged at each time point. The number of adherent cells per image was counted, and the average of the duplicate wells at each time point was calculated.

CACs were cultured for subsequent assessment of function via their capacity to adhere to a preformed layer of VSMC. Firstly, peripheral blood mononuclear cells were separated by Ficoll density gradient centrifugation of 30 ml of whole blood (Ficoll Paque PLUS, GE Healthcare, Buckinghamshire, UK). The remaining cells were suspended in endothelial growth medium (EGM; endothelial basal medium supplemented with 20% fetal cell serum, growth factors, and antibiotics; EBM‐2 Bullet kit; Lonza, Inx). Cells were cultured on 6‐well fibronectin‐coated plates (Millipore) at a density of 5 × 10^6^ per well and cultured for 7 days at 37°C in 5% CO_2_. On day 7, adherent CACs were detached from each well using a trypsin/EDTA solution. The CACs were isolated by centrifugation at 300 g for 10 min and adhesive capacity to VSMC examined as an index of function.

To perform the adhesion assay, confluent monolayers of VSMC (passage 2–6) were prepared in 96‐well plates. Cultured CACs harvested on day 7 (as described above) were seeded onto the VSMC monolayers at a density of 2.5 × 10^4^/well in 100 μl of EGM. Microscopic images at 100× magnification (five images per well) allowed the number of adherent CACs to the monolayers to be counted in duplicate wells after 10, 20, 30, 60, and 24 h post incubation. The variability in this technique of CAC adhesion to VSMC is high, with a coefficient of variation of 54% calculated for our laboratory.

### 2.9 Statistical analysis

Statistical analyses were performed using SPSS V.22 software. Data were assessed for normality using Shapiro Wilk. Log transformation was performed on non‐normal data (waist circumference, relative VO_2peak_, carotid‐brachial PWV, brachial‐foot PWV, peak SR, peak reactive hyperemia, TG, TC:HDL, and all variables related to the CAC number and adhesion), with variables subsequently back transformed and presented as geometric mean ± 95% confidence intervals. A one‐way ANOVA was used to identify if there were any group differences at pre‐training. A linear mixed model, with time (pre‐training vs. post‐training) and exercise group (MODERATE‐IT vs. HEAVY‐IT) treated as fixed factors, assessed the training responses. Following analysis of initial FMD results, the impact of pre‐training FMD upon post‐training FMD was established by splitting participants into normal FMD (>4.5%) or impaired FMD (<4.5%; FMD group), based on pre‐training FMD and irrespective of exercise training group, as described in Rossi et al. (2008). A separate linear mixed model was then used with time (pre‐training vs. post‐training), exercise group (MODERATE‐IT vs. HEAVY‐IT), and FMD group (normal vs. impaired) treated as fixed factors and relative FMD as the dependent variable. As NO bioavailability and CAC mobilization have been previously associated (Cubbon et al., 2010; Steiner et al., 2005), CAC number was also included as a dependent variable when comparing time, exercise group, and FMD group.

For the analysis of endothelial cell adhesion to VSMCs, a linear mixed model, with time (pre‐training vs. post‐training), exercise group (MODERATE‐IT vs. HEAVY‐IT), and time course as fixed factors, was performed. Subsequently, a one‐way ANOVA was used to interrogate the effect of exercise group (MODERATE‐IT vs. HEAVY‐IT) and time (pre‐training vs. post‐training) at each point in the time course.

Pearson correlations were performed to establish relationships between normally distributed variables. All normally distributed data were reported as mean ± SD; alpha was accepted at *p* < 0.05, exact P values are reported in tables, while in‐text *p* values are reported as *p* > 0.05 or *p* < 0.05. Paired t‐tests were used for post hoc analysis on variables where significant time by exercise group interactions was found. Cohen's d was used to determine effect sizes of non‐significant key variables from pre‐mean to post‐mean differences and pooled standard deviation in both exercise groups separately, only medium (0.5) and large (0.8) effects have been reported.

For a randomization into the MODERATE‐IT and HEAVY‐IT groups based on the primary outcome of FMD, a total of 34 participants were required, assuming an 80% power to detect an absolute change in FMD of 2% between groups with a standard deviation of 2% and an alpha of 0.05. Based on the secondary outcome of V̇O_2peak_, a total of 18 participants were required to obtain a power of 80% to detect a meaningful difference of 3 ml/kg/min between exercise groups, with a standard deviation of 2 ml/kg/min. Following completion of the study and based on our primary outcome of FMD, there was a 70% probability that the study would detect a significant treatment difference.

## 3 RESULTS

Participants successfully completed 99% of all laboratory‐based exercise sessions, with 70% of home‐based exercise training successfully completed during the 12‐week training period.

### 3.1 Cardiovascular and training parameters

**FIGURE 1:**
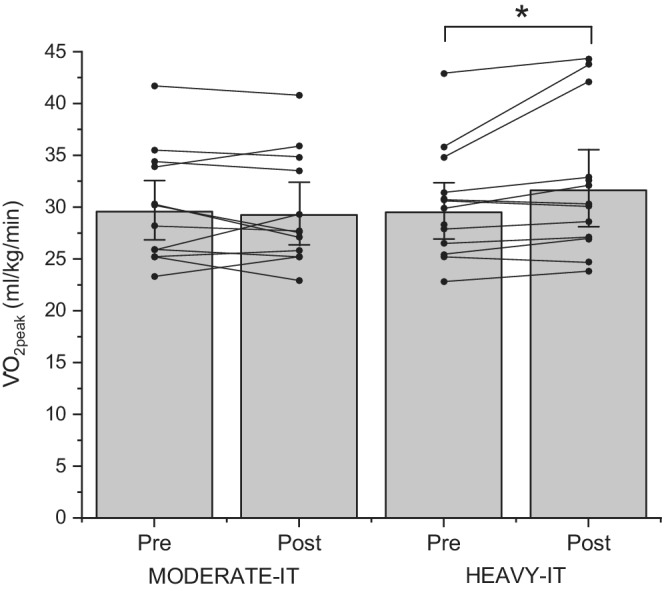
Individual participant (•) and mean data (gray bars) for relative V̇O_2peak_ which was unchanged following 12 weeks of MODERATE‐IT (*n* = 12; T‐test *p* = 0.59) but showed an increase * following HEAVY‐IT (*n* = 13; time effect *p* = 0.088; group effect *p* = 0.604; time x group effect *p* = 0.017; T‐test *p* = 0.02). Data presented as geometric mean ± 95% CI.

Participants did not differ in any variable prior to training (*p* > 0.05). Pre‐training and post‐training data are presented in Tables 1 and 2. The work rate during the work portion of the exercise protocols was 104 ± 24 W (63 ± 9% RISE WR_peak_) in the MODERATE‐IT exercise group and 133 ± 13 Watts (83 ± 6% RISE WR_peak_) in the HEAVY‐IT group. Following training, body mass decreased by 0.7 ± 1.4 kg and BMI by 0.3 ± 0.5 kg/m^2^, with no difference between exercise groups (time *p* < 0.05; time x exercise group *p* > 0.05). Waist circumference (time *p* > 0.05) showed a time x exercise group interaction (*p* < 0.05) with an increase pre‐training to post‐training in the MODERATE‐IT group (T‐test *p* < 0.05) but no difference in the HEAVY‐IT group (T‐test *p* > 0.05; Table 1). Waist–hip ratio showed a non‐significant, medium effect for a decrease with HEAVY‐IT training (effect size: HEAVY‐IT 0.60; MODERATE‐IT 0.39; time *p* > 0.05; Table 1). Absolute (Δ 0.09 ± 0.13 L/min; T‐test *p* < 0.05) and relative V̇O_2peak_ (Δ 2.4 ± 2.8 kg/ml/min; T‐test *p* < 0.05; Figure 1), GET (Δ 0.14 ± 0.15 L/min; T‐test *p* < 0.05), and WR_peak_ (Δ 14 ± 12 W; T‐test *p* < 0.05) all increased pre‐ to post‐HEAVY‐IT (time x exercise group *p* > 0.05) as reported in Table 1, whereas WR_peak_ increased to a lesser extent (Δ 5 ± 7 W; T‐test *p* < 0.05), and absolute (Δ –0.02 ± 0.13 L/min; T‐test *p* > 0.05), relative V̇O_2peak_ (Δ –0.4 ± 2.0 kg/ml/min; T‐test *p* > 0.05) and GET (Δ –0.02 ± 0.10 L/min; T‐test *p* > 0.05), were unchanged pre‐ to post‐MODERATE‐IT (time x exercise group *p* > 0.05; Table 1).

**TABLE 1:** Participant characteristics pre and post 12 weeks of MODERATE‐IT or HEAVY‐IT exercise training

	MODERATE‐IT (*n* = 13)		HEAVY‐IT (*n* = 14)		*p*‐values
	Pre	Post	Pre	Post	
Age (years)	55 ± 3	‐	57 ± 4	‐	Group = 0.210
Height (cm)	163 ± 4	‐	162 ± 6	‐	Group = 0.840
Body mass (kg)*	67.5 ± 9.7	67.6 ± 9.6	68.7 ± 12.2	67.5 ± 12.8	Time = 0.012 Group = 0.821 Interaction = 0.314
BMI (kg/m^2^)*	25.5 ± 3.4	25.6 ± 3.3	26.2 ± 5.4	25.8 ± 5.3	Time = 0.011 Group = 0.748 Interaction = 0.249
Waist circumference (cm)	81.3 (75.9–87.1)	81.7 (75.9–87.9)	84.0 (76.6–92.0)	80.4 (73.8–87.5)	Time = 0.283 Group = 0.885 Interaction = 0.028 T‐test MODERATE‐IT *p* = 0.04 T‐test HEAVY‐IT *p* = 0.09
WHR†	0.81 ± 0.06	0.78 ± 0.06	0.82 ± 0.06	0.79 ± 0.04	Time = 0.072 Group = 0.929 Interaction = 0.422
SBP (mmHg)	117 ± 12	114 ± 9	117 ± 12	114 ± 12	Time = 0.123 Group = 0.979 Interaction = 0.823
DBP (mmHg)	76 ± 6	74 ± 7	73 ± 7	74 ± 8	Time = 0.487 Group = 0.587 Interaction = 0.275
MAP (mmHg)	90 ± 8	87 ± 7	88 ± 8	87 ± 9	Time = 0.274 Group = 0.766 Interaction = 0.417
PP (mmHg)	41 ± 8	40 ± 10	44 ± 6	41 ± 6	Time = 0.139 Group = 0.598 Interaction = 0.349
Absolute V̇O_2peak_ (L/min)ǂ	1.99 ± 0.27	1.98 ± 0.32	2.01 ± 0.24	2.11 ± 0.27	Time = 0.132 Group = 0.452 Interaction = 0.029 T‐test MODERATE‐IT *p* = 0.63 T‐test HEAVY‐IT *p* = 0.02
GET (L/min)*,ǂ	1.13 ± 0.19	1.11 ± 0.17	1.08 ± 0.15	1.22 ± 0.17	Time = 0.023 Group = 0.628 Interaction = 0.003 T‐test MODERATE‐IT *p* = 0.50 T‐test HEAVY‐IT *p* = 0.01
WR_peak_ (W)*,ǂ	165 ± 20	170 ± 23	158 ± 17	172 ± 21	Time = 0.001 Group = 0.721 Interaction = 0.037 T‐test MODERATE‐IT = 0.04 T‐test HEAVY‐IT *p* = 0.01
HR_peak_ (bpm)	169 ± 10	169 ± 9	166 ± 9	164 ± 12	Time = 0.106 Group = 0.262 Interaction = 0.305

*Notes*: Data are mean ± SD or geometric mean (95% CI) for variables that were log transformed.

Abbreviations: BMI, body mass index; DBP, diastolic blood pressure; GET, gas exchange threshold; HR, heart rate; MAP, mean arterial pressure; PP, pulse pressure; SBP, systolic blood pressure; WHR, waist–hip ratio; WR, work rate.

* Significant time effect (*p* < 0.05).

ǂ Significant time by group interaction (*p* < 0.05).

† Medium effect size in HEAVY‐IT group (Cohen's d).

**TABLE 2:** Variables associated with the FMD procedure, PWV between sites in the arterial tree and blood biomarkers assessed pre and post 12 weeks of exercise training in both the MODERATE‐IT and HEAVY‐IT groups

	MODERATE‐IT (*n* = 13)		HEAVY‐IT (*n* = 12)		*p*‐values
	Pre	Post	Pre	Post	
Baseline diameter (mm)	3.65 ± 0.29	3.69 ± 0.28	3.70 ± 0.48	3.67 ± 0.49	Time = 0.910 Group = 0.922 Interaction = 0.089
Peak diameter (mm)	3.83 ± 0.32	3.87 ± 0.29	3.88 ± 0.50	3.82 ± 0.51	Time = 0.690 Group = 0.997 Interaction = 0.078
Absolute FMD (mm)	0.18 ± 0.08	0.18 ± 0.11	0.19 ± 0.11	0.15 ± 0.07	Time = 0.482 Group = 0.780 Interaction = 0.340
Relative FMD (%)	4.88 ± 2.21	5.03 ± 2.88	4.78 ± 2.55	4.16 ± 2.02	Time = 0.710 Group = 0.500 Interaction = 0.536
AUC_60_ (a.u.)	32,064 ± 12,885	31,208 ± 9185	33,529 ± 13,287	28,421 ± 7832	Time = 0.287 Group = 0.764 Interaction = 0.553
AUC_90_ (a.u.)	42,477 ± 17,143	40,136 ± 10,524	42,294 ± 16,424	34,765 ± 9338	Time = 0.159 Group = 0.474 Interaction = 0.580
Peak SR (s^−1^)	1718 (1462–2018)	1667 (1380–2014)	1648 (1291–2104)	1866 (1535–2270)	Time = 0.580 Group = 0.515 Interaction = 0.443
Peak hyperemia (cm/s)	64.6 (44.3–94.2)	76.6 (63.5–92.3)	77.1 (63.4–93.8)	84.7 (72.6–98.86)	Time = 0.074 Group = 0.382 Interaction = 0.788
Carotid‐Brachial PWV (m/s)*	6.49 (5.62–7.48)	6.12 (5.30–7.08)	6.30 (5.77–6.87)	5.14 (3.92–6.75)	Time = 0.048 Group = 0.275 Interaction = 0.312
Brachial‐Foot PWV (m/s)	7.78 (7.10–8.53)	8.55 (7.98–9.16)	7.93 (6.93–9.06)	8.71 (7.73–9.82)	Time = 0.055 Group = 0.716 Interaction = 0.968
LDL (mmol/L)	2.76 ± 1.45	2.84 ± 1.53	3.44 ± 0.67	3.56 ± 0.65	Time = 0.187 Group = 0.107 Interaction = 0.736
HDL (mmol/L)	1.83 ± 0.33	1.89 ± 0.42	2.0 ± 0.57	2.12 ± 0.62	Time = 0.056 Group = 0.310 Interaction = 0.469
TG (mmol/L)ǂ	1.00 (0.84–1.18)	0.89 (0.73–1.09)	0.92 (0.75–1.12)	0.97 (0.78–1.20)	Time = 0.393 Group = 0.989 Interaction = 0.032 T‐test MODERATE‐IT = 0.09 T‐test HEAVY‐IT = 0.30
TC (mmol/L)	5.46 ± 0.93	5.56 ± 1.00	5.76 ± 0.82	6.05 ± 0.91	Time = 0.084 Group = 0.247 Interaction = 0.362
TC:HDL	2.50 (1.71–3.65)	3.15 (2.67–3.72)	3.12 (2.75–3.53)	3.12 (2.71–3.59)	Time = 0.109 Group = 0.657 Interaction = 0.162
ET‐1 (pg/ml)	1.53 ± 0.40	1.65 ± 0.38	1.58 ± 0.45	1.75 ± 0.38	Time = 0.065 Group = 0.581 Interaction = 0.771

*Notes*: Data are presented as mean ± SD or geometric mean (95% CI) for variables that were log transformed.

Abbreviations: AUC, area under the curve; ET‐1, endothelin‐1; FMD, flow‐mediated dilation; HDL, high‐density lipoprotein; LDL, low‐density lipoprotein; PWV, pulse wave velocity; SR, shear rate; TC, total cholesterol; TG, triglyceride.

* Significant time effect (*p* < 0.05).

ǂ Significant time by group interaction (*p* < 0.05).

Blood pressure (Table 1) and absolute and relative FMD (effect size: HEAVY‐IT absolute 0.46, relative 0.27; MODERATE‐IT absolute 0.06, relative 0.06; Figure 4a) including the hyperemic and shear stimuli for FMD were unaffected by exercise training (time *p* > 0.05; Table 2). When participants were grouped into normal versus impaired endothelial function based on pre‐training FMD, participants with impaired endothelial function demonstrated an increase in FMD following exercise (Δ 1.80 ± 2.39%; time x FMD group *p* < 0.05; T‐test *p* < 0.05), whereas participants with a normal FMD response pre‐training demonstrated a decrease in FMD following training (Δ –2.07 ± 2.43%; time x FMD group *p* < 0.05; T‐test *p* < 0.05), irrespective of whether the exercise training was MODERATE‐IT or HEAVY‐IT (time x FMD group x exercise group *p* > 0.05).

**FIGURE 2:**
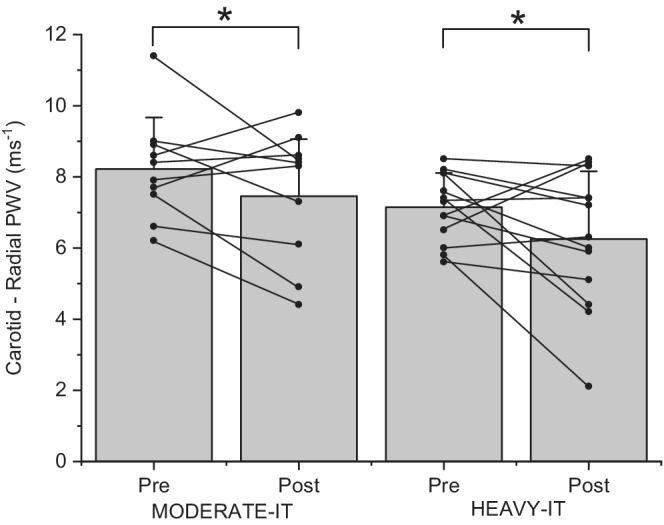
Individual participant (•) and mean data (gray bars) for carotid radial pulse wave velocity which decreased over the course of the 12‐week exercise intervention, irrespective of exercise group (MODERATE‐IT *n* = 10; HEAVY‐IT *n* = 13; time effect *p* = 0.018 *; group effect *p* = 0.042; time x group interaction *p* = 0.505).

Carotid‐radial (Figure 2) and carotid‐brachial PWV decreased by 7 ± 21% and 8 ± 12%, respectively, irrespective of exercise intensity (time *p* < 0.05; Table 2). Brachial‐foot PWV was unchanged (time *p* > 0.05). Exercise training at either intensity did not affect LDL, HDL, TC, TC:HDL, or ET‐1 concentrations (ET‐1 effect size: HEAVY‐IT 0.42; MODERATE‐IT 0.35; *p* > 0.05; Table 2). TG concentration demonstrated a time x exercise group interaction (*p* < 0.05) but no differences pre‐training to post‐training in either group (time effect *p* > 0.05; group effect *p* > 0.05; T‐test *p* > 0.05; effect size: HEAVY‐IT 0.13; MODERATE‐IT 0.37; Table 2).

### 3.2 Circulating angiogenic cell number and function

**FIGURE 3:**
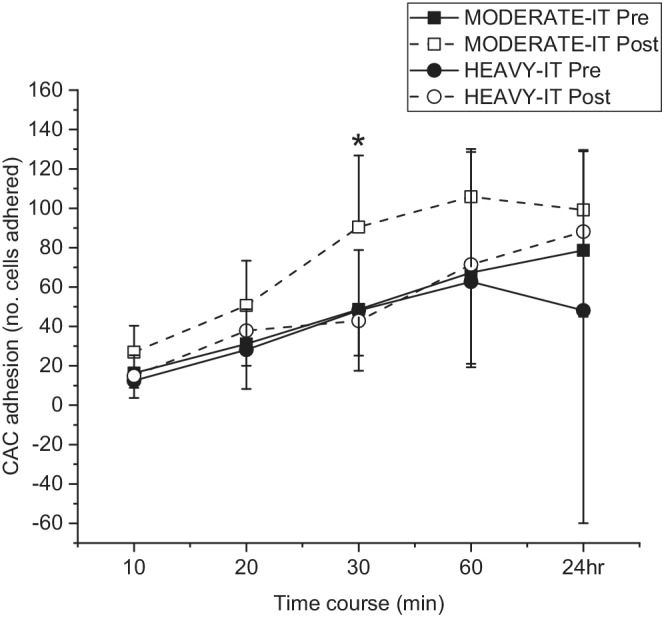
The time course of CAC adhesion to vascular smooth muscle cells pre (closed symbols) and post (open symbols) the 12‐week exercise intervention in the MODERATE‐IT (square) and HEAVY‐IT (circle) groups. Adhesion of CACs to VSMC increased over 24 h of the adhesion protocol in both groups pre‐training and post‐training (time effect *p* = 0.083; group effect *p* = 0.372; time course *p* = 0.001). There was no change in adhesion from pre‐training to post‐training in the HEAVY‐IT group at any time point (*n* = 7; T‐Test *p* > 0.05), however adhesion was increased at 30 min post training in the MODERATE‐IT group (*n* = 6) compared to pre‐training (T‐Test *p* = 0.06).

The number of CD34^+^, CD34^+^/CD45^dim^, and CD34^+^/CD45^dim^/KDR^+^ CACs was unchanged by exercise training (*p* > 0.05; Table 3). Impaired or normal endothelial function prior to training also had no effect on CAC number in response to exercise training (*p* > 0.05). However, the adhesive capacity of CACs to VSMC following culture increased over a 24 h period (time course *p* < 0.05; Figure 3). In the HEAVY‐IT group, this adhesive capacity did not change from pre‐training to post‐training. However, in the MODERATE‐IT group, the number of CACs adhering to VSMC 30 min following plating was greater post‐training compared to pre‐training (time *p* < 0.05; Figure 3).

**TABLE 3:** Numbers of CAC pre and post 12 weeks of exercise training in the MODERATE‐IT and HEAVY‐IT groups

	MODERATE‐IT (*n* = 11)		HEAVY‐IT (*n* = 12)		Significance
	Pre	Post	Pre	Post	
CD34^+^ per 100,000 leukocytes	20.80 (13.77–31.41)	18.66 (12.97–26.85)	16.83 (14.32–19.77)	15.67 (10.52–23.33)	Time = 0.539 Group = 0.261 Interaction = 0.902
CD34^+^/CD45^dim^ per 100,000 leukocytesǂ	4.70 (2.79–7.93)	6.37 (3.40–11.91)	6.78 (5.41–8.49)	4.84 (3.15–7.45)	Time = 0.921 Group = 0.856 Interaction = 0.061
CD34^+^/CD45^dim^/KDR^+^ per 100,000 leukocytes	0.18 (0.08–0.39)	0.19 (0.07–0.52)	0.25 (0.09–0.64)	0.12 (0.07–0.18)	Time = 0.463 Group = 0.346 Interaction = 0.297

*Notes*: Data are presented as geometric mean (95% CI) as variables were log transformed.

ǂ Significant time by group interaction (*p* < 0.05).

## 4 DISCUSSION

To our knowledge, this is the first study to compare the effect of interval exercise training of differing intensities upon markers of cardiovascular health in postmenopausal females. The main findings were that only HEAVY‐IT increased V̇O_2peak_ and GET, while only MODERATE‐IT increased CAC adhesion to VSMC. Additionally, peripheral pulse wave velocity decreased with exercise training irrespective of group, while endothelial function improved with exercise training in participants who had impaired endothelial function at pre‐training, irrespective of exercise group. Standard blood biomarkers of cardiovascular disease (i.e., LDL, HDL, and TC) were unchanged following the exercise training period.

### 4.1 Traditional cardiovascular disease risk factors were not affected by exercise training

The prevalence of obesity increases with age and is a significant factor in traditional CVD risk (Kaminsky et al., 2013). Body mass and BMI were reduced following exercise training, irrespective of exercise group, in the current study (Table 1). BMI prior to exercise training was in the healthy to overweight category; therefore, reductions in BMI toward a healthier range are important. The menopausal transition leads to lower estrogen but higher testosterone levels resulting in greater fat deposition around the waist. These changes in waist circumference occur independently of changes in body mass (Church et al., 2009), and while body mass decreased with exercise training, waist circumference did not change. However, WHR showed a non‐significant, medium effect for a decrease following HEAVY‐IT but not MODERATE‐IT training. Both exercise groups prior to training had a WHR of ≤0.82, which is categorized as low to moderate risk of CVD. Interval exercise training within either the moderate or heavy‐intensity domains is effective for reducing body mass, whereas more research is required to determine whether heavy‐intensity intervals are more effective than moderate intensity for alterations in body composition.

Circulating lipid/lipoproteins (e.g., LDL, HDL, and TC) as indices of CVD risk were not altered in either exercise group, with the exception of TG, which showed a significant time by group interaction but no significant differences, and small effect sizes, pre‐training to post‐training in either group (Table 2). Values for all of these biomarkers ranged from optimal to borderline high, even after training (NCEP, 2001), while TG concentration remained well within the normal range pre‐training and post‐training. Previous studies have shown that changes in lipid concentrations are dependent upon reductions in adiposity requiring energy deficits brought about through large volumes of exercise training (Stewart et al., 2005). Indeed, a meta‐analysis suggested that a large decrease in body weight was required for favorable effects on the entire lipid/lipoprotein profile (Leon & Sanchez, 2001). The small reduction in BMI and unchanged WHR suggest that adiposity was not altered enough via these training regimes to impact the lipid/lipoprotein profile in the present study.

### 4.2 Heavy‐intensity interval training was more effective for improving V̇O_2peak_

Cross‐sectional studies have suggested that postmenopausal females have lower V̇O_2peak_ compared to pre‐menopausal females (Mercuro et al., 2006), although exercise training has been shown to be effective at improving V̇O_2peak_ following the menopause (reviewed by Asikainen et al. (Asikainen et al., 2004)). A 1 MET (equivalent to a V̇O_2_ of 3.5 ml/kg/min) increase in V̇O_2peak_ has been associated with a 13%–15% reduction in all‐cause mortality and CVD (Kodama et al., 2009). In the present study, there was an 8% (2.4 ± 2.8 ml/kg/min) increase in relative V̇O_2peak_ following HEAVY‐IT, with no change following MODERATE‐IT. This suggests an indicative reduction in all‐cause mortality and CVD of approximately 9%–10% on average following 12 weeks of HEAVY‐IT. Previous literature indicates that increases in V̇O_2peak_ and GET are training intensity, rather than volume dependent (Kemi et al., 2005; Rakobowchuk, Harris, Taylor, Cubbon, & Birch, 2012). Consistent with this, our results suggest that performing interval exercise in a manner that increases the intensity (e.g., V̇O_2_ equivalent to 90% GET vs. 120% GET) is efficacious for increasing V̇O_2peak_ in postmenopausal females.

### 4.3 Exercise training improved impaired endothelial function

Endothelial function, assessed via FMD, is reduced in postmenopausal compared to pre‐menopausal females (Holder et al., 2019). Exercise has previously shown to be effective at improving endothelial function via shear stress‐mediated increases in NO bioavailability (Green et al., 2004). We hypothesized that HEAVY‐IT would elicit a greater shear stress response than MODERATE‐IT due to the increased cardiometabolic demands. This would result in greater NO production and therefore improved post hyperemic vasodilation during FMD following exercise training. However, endothelial function was unchanged across the 12 weeks of exercise training in both groups. The lack of an observed change was perhaps due to the time course of FMD assessments. Changes in endothelial function with exercise training have been reported to occur in the first 2–6 weeks, with the same study reporting the improvement in endothelial function was lost at 8–12 weeks (Tinken et al., 2008), suggesting any improvements may have been missed in the present study. Previous exercise studies in postmenopausal females have also shown no improvement in brachial artery endothelial function after 12 weeks (Swift et al., 2014), and in popliteal artery endothelial function after 8 weeks of exercise training (Hoier et al., 2021). The lack of improvement in endothelial function following longer duration exercise training has been suggested to be a consequence of structural adaptations resulting in increased arterial diameter with a smaller vasodilation during FMD (Tinken et al., 2008). In this instance, an increase in resting brachial artery diameter would be observed, which was not evidenced in the present study.

**FIGURE 4:**
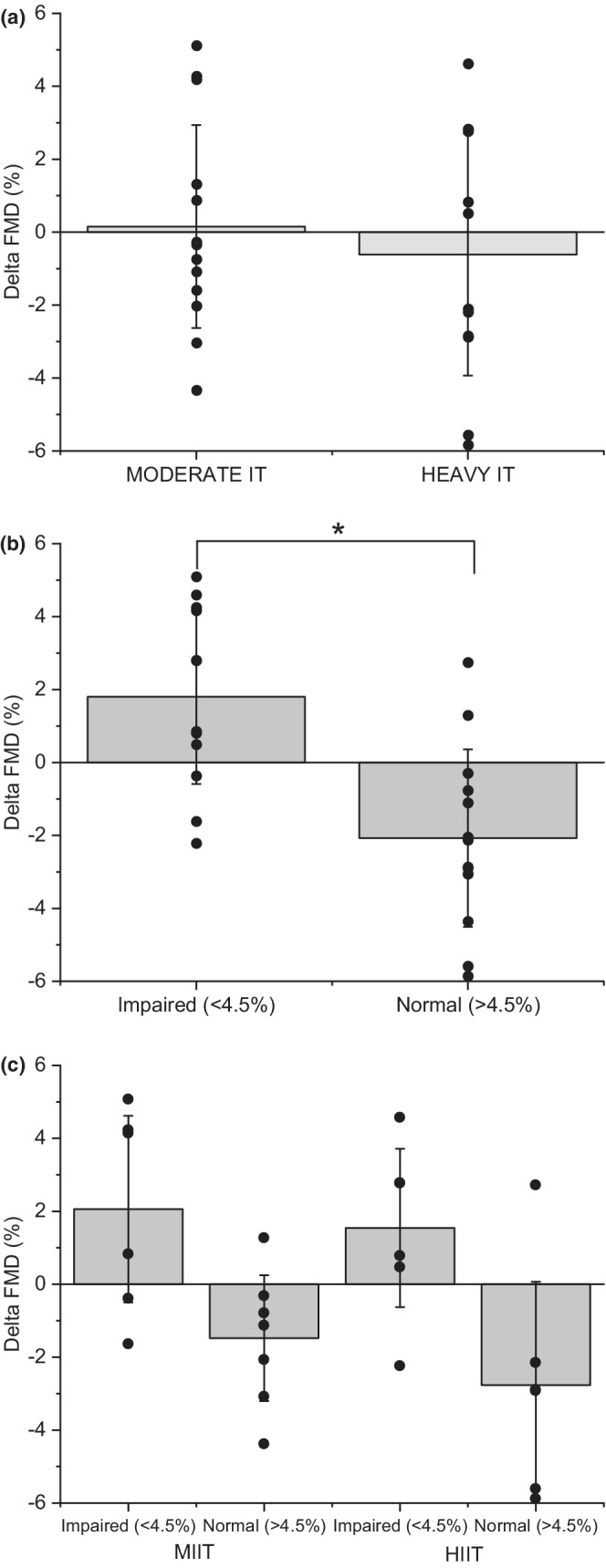
(a) There was no difference in relative FMD (%) from pre to post 12 weeks of exercise training in the MODERATE‐IT and HEAVY‐IT groups (time x exercise group *p* > 0.05). (b) Pre‐training, relative FMD was determined to be impaired (<4.5%) or normal (>4.5%) across both exercise groups, as per Rossi et al. (Rossi et al., 2008). FMD increased from pre‐training to post‐training, irrespective of exercise group, in participants with impaired FMD (T‐test *p* < 0.05), but decreased with training in participants who started the study with normal FMD (T‐test *p* < 0.05; Time x FMD group *p* < 0.05). (c) Impaired FMD prior to exercise training is a greater determinant of whether FMD improved following exercise training compared to the intensity of exercise training prescribed which appeared to have no effect (time × FMD group × exercise *p* > 0.05).

A further possibility is that participants who had a normal FMD prior to exercise training did not respond to this intervention compared to participants with an impaired FMD prior to exercise training. In previous studies, a cutoff value of 4.5% for relative FMD has been used to categorize postmenopausal females as either having impaired (<4.5%) or normal FMD (>4.5%) responses (Rossi et al., 2008; Swift et al., 2014). Upon further investigation of our data, both the mean (4.8 ± 2.3%; Table 2) and median (4.7%, IQR 3.4–6.4%) relative FMD prior to exercise training were close to the cutoff of 4.5%. When using 4.5% to separate participants prior to exercise training into impaired or normal FMD groups, consistent with previous studies (Rossi et al., 2008; Swift et al., 2014), postmenopausal females with impaired FMD prior to training (*n* = 12) had improvements in FMD following training, while in the group with a normal FMD prior to training (*n* = 13), there was no change in FMD following training (Figure 4b). This is consistent with previous exercise training studies in postmenopausal females with impaired FMD prior to the training period (Swift et al., 2014). When we factored in the effect of exercise intensity, that is, MODERATE‐IT versus HEAVY‐IT impaired FMD prior to training was a more important factor for determining whether exercise training would be effective at improving FMD with exercise intensity having less influence (Figure 4c). Therefore, any intensity of exercise training may be effective at improving endothelial function in postmenopausal females with impaired FMD. While for postmenopausal females with normal FMD, exercise training may be useful for preventing age‐related declines and preserving a healthy endothelial function, however this has not yet been shown and requires further investigation.

A greater volume of shear stress may be required to sufficiently increase NO bioavailability for the improvement of endothelial function in postmenopausal females where FMD prior to training is not impaired. In the present study, HEAVY‐IT was prescribed at 120% GET, the lower end of the heavy‐intensity exercise domain, which gives scope to increase the demands of the exercise, while still remaining tolerable and within the heavy‐intensity domain. This would elicit higher heart rates, in turn increasing the volume of shear stress accumulated during the training session.

### 4.4 Peripheral arterial stiffness improved with exercise training

A meta‐analysis by Ashor et al. (2014) indicated that aerobic exercise training produced greater reductions in peripheral compared to central arterial stiffness, with larger reductions observed following more than 10 weeks of aerobic exercise training. Additionally, a greater exercise intensity was associated with greater reductions in arterial stiffness (Ashor et al., 2014). In the current study, peripheral arterial stiffness (carotid‐radial and carotid‐brachial PWV) decreased following training in both groups, irrespective of exercise intensity. The lack of difference observed between the MODERATE‐IT and HEAVY‐IT exercise groups may be due to matching exercise volume and the number of exercise training sessions (i.e., total work done). While the two exercise protocols were within different exercise intensity domains, the mean difference in the workload during exercise training may not have been sufficiently large enough to induce a difference in peripheral PWV (HEAVY‐IT 83% RISE WR_peak_ vs. MODERATE‐IT 63% RISE WR_peak_; *p* < 0.05). Indeed, it is likely that changes in peripheral PWV are dependent upon heart rate and blood pressure during exercise, perhaps in the present study, heart rate and blood pressure were not of sufficiently different magnitudes to differentially alter peripheral PWV.

The reduction in peripheral arterial stiffness in both exercise training groups in the present study is interesting considering the lack of change in brachial artery FMD and blood pressure. Exercise‐induced shear stress increases NO bioavailability, improving both endothelial function and arterial stiffness (Joyner, 2000). As we did not observe any improvement in FMD, we cannot ascertain whether NO bioavailability was increased following training. Systolic and diastolic blood pressure and MAP were within a healthy range prior to the exercise intervention and were unchanged following training. Arterial stiffness has been associated with chronic low‐grade inflammation in previous studies, specifically CRP, IL‐6, and TNF‐α (Booth et al., 2004; Moreau et al., 2013). While inflammatory markers were not assessed in the present study, it is possible that both exercise training interventions reduced inflammation resulting in reductions in peripheral PWV. Additionally, reduction in the vasoconstrictor ET‐1 has a potent role in endothelial dysfunction (Donato et al., 2009) and arterial stiffness (McEniery et al., 2003), however in the present study, there was no change in ET‐1 following exercise training. Alternative mechanisms including alterations in other vasoconstrictors such as angiotensin and prostaglandins, the repair of elastic fibers, and the breaking of collagen cross‐linking (Tanaka & Safar, 2005) may be responsible for the observed changes in peripheral arterial stiffness.

While carotid‐radial and carotid‐brachial PWV reduced in response to exercise training in the present study, brachial‐foot PWV was unchanged. Perhaps, in future studies with increased statistical power, the changes in brachial‐foot PWV may become significant. The reductions in peripheral arterial stiffness in postmenopausal females with exercise training in the present study are important as menopause has been shown as an independent factor contributing to age‐related increases in arterial stiffness (Zaydun et al., 2006). While in females, heart failure with preserved ejection fraction and coronary microvascular dysfunction are more commonly diagnosed forms of cardiovascular disease and are associated with increased arterial stiffening in females (Beale et al., 2018; Coutinho et al., 2013; Coutinho et al., 2017).

### 4.5 MODERATE‐IT improved circulating angiogenic cell adhesive function

Postmenopausal females have lower numbers of CD34+KDR+ and CD34+CD133+KDR+ compared to pre‐menopausal females (Bulut et al., 2007), likely through downregulation of the eNOS pathway following loss of estrogen. The present study showed no change in CACs following 12 weeks of exercise training in postmenopausal females. Previously, our laboratory has shown an increase in CAC number (CD34^+^) in healthy females following 4 weeks of sprint interval or continuous exercise training (Harris et al., 2014). NO appears to have an essential role in exercise‐induced CAC mobilization as when NO production is blocked in humans, CAC mobilization in response to acute exercise is abolished (Cubbon et al., 2010). Additionally, CAC mobilization has been correlated with surrogates of NO bioavailability in humans (Steiner et al., 2005).

We hypothesized that HEAVY‐IT would elicit greater shear stress due to greater blood flow and cardiac demands, that would increase NO bioavailability that would also produce greater CAC mobilization compared to MODERATE‐IT. In contrast to our hypothesis, FMD was unchanged with exercise training suggesting no increase in NO bioavailability, while CAC number also did not change. This may suggest that the exercise training intervention in the present study was not effective at increasing NO production resulting in no increase in CACs. Alternatively as discussed earlier, only postmenopausal females with impaired endothelial function demonstrated improvements in FMD following exercise training. Therefore, we assessed whether postmenopausal females with impaired endothelial function also showed improvements in CAC number following exercise training, compared to postmenopausal females with normal endothelial function prior to training. No difference in CACs was found between participants with impaired or normal endothelial function and the response to the exercise training was the same, that is, no change in CAC number.

We also explored the adhesive capacity of CACs to VSMCs in cell culture, the purpose of which was to mimic in vitro, the scenario of CAC activity that may occur in vivo in endothelial repair mechanisms of the arterial wall. A previous study injected human CACs, collected following exercise training, into mice with induced carotid artery injury which resulted in improved re‐endothelialization compared to the non‐exercise human control group (Sonnenschein et al., 2011). While CAC adhesion to VSMCs increased over the course of 24 h in both exercise groups in the present study, there was no difference in adhesion pre‐exercise to post‐exercise training in the HEAVY‐IT group. In contrast, the MODERATE‐IT group showed enhanced adhesive capacity of CACs post‐training, particularly at 30 min into the time course. This suggests that the adhesive capacity of CACs improves the longer they are exposed to the VSMCs and that this process is improved following MODERATE‐IT but not HEAVY‐IT. Moderate‐intensity exercise may be more beneficial for improving CAC function in the same way MODERATE‐IT appears more beneficial for increasing CAC number. In this population of postmenopausal females, MODERATE‐IT may have been more effective at reducing inflammation compared to HEAVY‐IT. Inflammation has been suggested to impair the reparative function of CACs, through reduced expression of E‐selectin and integrin α_v_β_5_, thus impairing adhesion (Di Santo et al., 2008). Therefore, MODERATE‐IT could have reduced chronic inflammation in these postmenopausal females, thus enabling increased expression of adhesion molecules to better allow CACs to adhere to VSMCs in vitro.

Future studies should explore whether the expression of the aforementioned adhesion molecules is downregulated in postmenopausal compared to pre‐menopausal females and whether exercise training is capable of upregulating adhesion molecule expression. Additionally, we have suggested that inflammation may be a key factor in altering the ability of the endothelium to repair, therefore future studies should quantify inflammatory molecules, such as IL‐1, IL‐6, and TNFα, in postmenopausal females and then assess the effect of exercise training and the association with any changes in CAC number or adhesive function.

### 4.6 Study limitations

In this study, we assessed CACs, as biomarkers to indicate whether the reparative capacity of endothelial cells was improved following exercise training of different intensities. However, we recognize that CACs with surface markers CD34^+^/ KDR^+^/ CD45^dim^ cannot be definitively confirmed as endothelial specific and may be more global reparative markers. Indeed, the role of these cells in cardiovascular physiology is not clear as most circulating adhesion molecules are considered to be adverse due to their involvement in plaque formation. Nevertheless, CD34^+^ cells are hematopoietic stem cells which are mobilized in response to vascular injury to aid repair (Rehman et al., 2003), as shown in an ischemic hind‐limb mouse model (Asahara et al., 1997). In addition to CD34^+^, the inclusion of CD45^dim^ and KDR^+^ cells has been suggested to be the best compromise for quantifying circulating angiogenic cells with acceptable levels of sensitivity, specificity, and reliability (Fadini et al., 2012). However, further work is required to refine and characterize these markers in humans, particularly following exercise interventions.

As highlighted by our power analysis, the study was powered to detect a statistically significant difference in V̇O_2peak_ but was slightly underpowered to detect a significant difference in FMD between the MODERATE‐IT and HEAVY‐IT groups. Due to the standard deviation in the variables related to CAC number and adhesive function, it is likely the study was underpowered to detect significant differences between the two exercise groups over the training period. The sample sizes for CAC number and adhesive function were also reduced, further affecting the power of the current study, and were due to venipuncture failure when assessing CAC number and issues during the culturing of CACs prior to the adhesion experiments. Additionally, as per the design of the study, work rates were selected which in a continuous manner would increase the intensity of the exercise and make it less sustainable compared to exercising at these work rates for brief periods, as with interval exercise. However, interval exercise at the prescribed exercise intensities, may not have generated sufficient energy expenditure to induce adaptations in some of the cardiovascular markers selected in the present study.

This study aimed to delineate the effect of exercise intensity during interval exercise upon markers of cardiovascular health in postmenopausal females; therefore, exercise intensity and frequency were controlled, however duration of the exercise sessions was greater in the MODERATE‐IT group to control for total work between both groups. Therefore, while differences in cardiovascular markers between MODERATE‐IT and HEAVY‐IT groups are likely dependent upon the differences in exercise intensity, the effect of differences in exercise duration cannot be eliminated.

## 5 CONCLUSIONS

HEAVY‐IT appears to be the most effective mode and intensity of exercise when targeting improvements in V̇O_2peak_ in a population of postmenopausal females. In contrast, the ability of circulating angiogenic cells to adhere to vascular smooth muscle cells is improved following MODERATE‐IT and not HEAVY‐IT. Interval exercise training reduced peripheral arterial stiffness and improved FMD in postmenopausal females with impaired endothelial function, while markers of blood pressure, LDL, and HDL were unchanged, regardless of exercise intensity.

## AUTHOR CONTRIBUTIONS

G.K.L., G.K.B., C.F., K.E.P., and K.M.B. designed these experiments. The article was written by G.K.L. with editorial input from K.M.B., K.E.P., E.H., G.K.B., K.R‐S., M.T.K., and C.F. Experimental research was carried out by G.K.L., G.K.B., and E.H. and analyzed by G.K.L., G.K.B. and K.M.B. K.E.P. and K.R‐S. advised on all cell experiments.

## FUNDING INFORMATION

Funded by the Dunhill Medical Trust.
